# Supplementary material for: Nrf2 Downregulation Contributes to Epithelial-to-Mesenchymal Transition in Helicobacter pylori-Infected Cells
Source: Cancers (Basel). 2022 Sep 2;14(17):4316. doi: 10.3390/cancers14174316 (PMC9455077; doi:10.3390/cancers14174316)
Supplement: Supplementary file 1 [file cancers-14-04316-s001.zip › cancers-1853650-supplementary.pdf]

Supplementary Materials and Results

SUPPLEMENTARY FIGURES

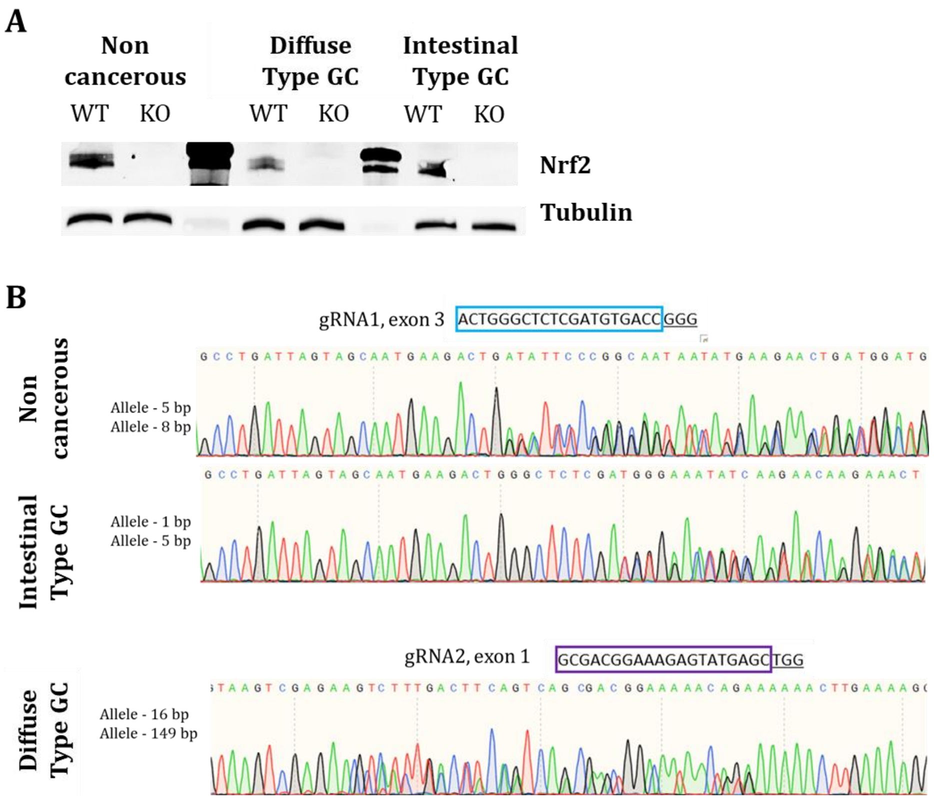

Figure S1. Verification of Nrf2 invalidation by CRISPR-cas9 in the three gastric cell lines. (**A**) The invalidation of Nrf2 was assessed by western-blotting. (**B**) The invalidation of Nrf2 was assessed by sequencing.

A

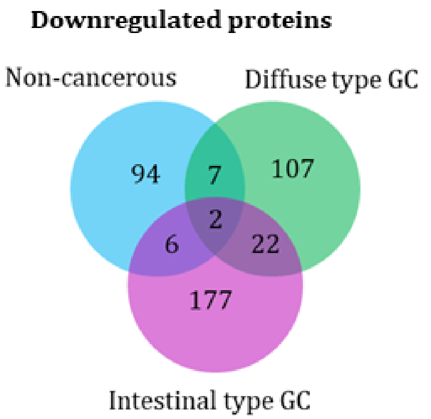

C

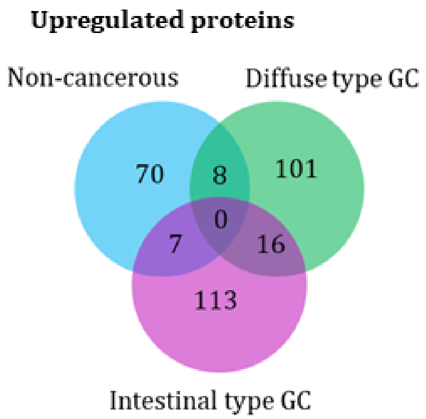

B

Common downregulated proteins			
**Cell lines**	**Total protein number**	**Uniprot ID**	**Protein ID**
Non cancerous<br>Diffuse type GC<br>Intestinal type GC	2	B4DLR8	NQO1
		A0A3B3IRX2	
Non cancerous<br>Diffuse type GC	7	Q7L5L3	GDPD3
		Q92764	KRT35
		A0A3B3ISS6	
		A0A0G2JMB2	
		Q9UBC9	SPRR3
		M0QXX2	
		Q96GW9	SYMM
Non cancerous<br>Intestinal type GC	6	Q9Y2K2	SIK3
		Q8N1P7	CRBG2
		H3BNQ7	
		Q86UY8	NT5D3
		O75368	SH3L1
		O15400	STX7
Diffuse type GC<br>Intestinal type GC	22	P09913	FIT2
		P48163	MAOX
		O75342	LX12B
		Q9UM01	YLAT1
		Q5T750	XP32
		P48507	GSH0
		F6Y5H0	
		Q5T749	KPRP
		A0A1B0GVI3	
		Q15120	PDK3
		Q49AR2	CE022
		Q15118	PDK1
		P01037	CYTN
		R4GN49	
		Q96B97	SH3K1
		Q8N3D4	EH1L1
		Q13835	PKP1
		P20930	FILA
		Q9NYJ8	TAB2
		P23490	LORI
		Q5D862	FILA2
		Q6KB66	K2C80

D

Common upregulated proteins			
**Cell lines**	**Total protein number**	**Uniprot ID**	**Protein ID**
Non cancerous<br>Diffuse type GC<br>Intestinal type GC	0		
Non cancerous<br>Diffuse type GC	8	Q99747	SNAG
		A0A2R8Y566	
		Q14194	DPYL1
		P00966	ASSY
		O00189	AP4M1
		Q9HC84	MUC5B
		P48668	K2C6C
		Q562R1	ACTBL
Non cancerous<br>Intestinal type GC	7	P04279	SEMG1
		Q9BXW6	OSBL1
		Q9Y6Y0	NS1BP
		O95678	K2C75
		Q12834	CDC20
		P15941	MUC1
		Q8TAP6	CEP76
Diffuse type GC<br>Intestinal type GC	22	A8MPP1	D11L8
		Q7L775	EPMIP
		Q86SX6	GLRX5
		Q5TAW7	
		Q52LG2	KR132
		Q3B726	RPA43
		P04083	ANXA1
		Q6PL18	ATAD2
		Q12789	TF3C1
		Q9NW82	WDR70
		J3KNR0	
		E5RIH5	
		A8CG34	P121C
		Q9Y6W3	CAN7
		Q15910	EZH2
		Q8IX90	SKA3

Figure S2. Common regulated proteins in the three Nrf2-KO *vs* WT gastric cell lines. (**A**) Venn diagram representing overlaps among the differentially down-regulated (p-value <0.05; abundance ratio <0.65) identified in the three different Nrf2-KO cell lines. (**B**) List of the common down-regulated proteins between the three cell lines. (**C**) Venn diagram representing overlaps among the differentially up-regulated (p-value <0.05; abundance ratio >1.5) identified in the three different Nrf2-KO cell lines. (**D**) List of the common up-regulated proteins between the three cell lines.

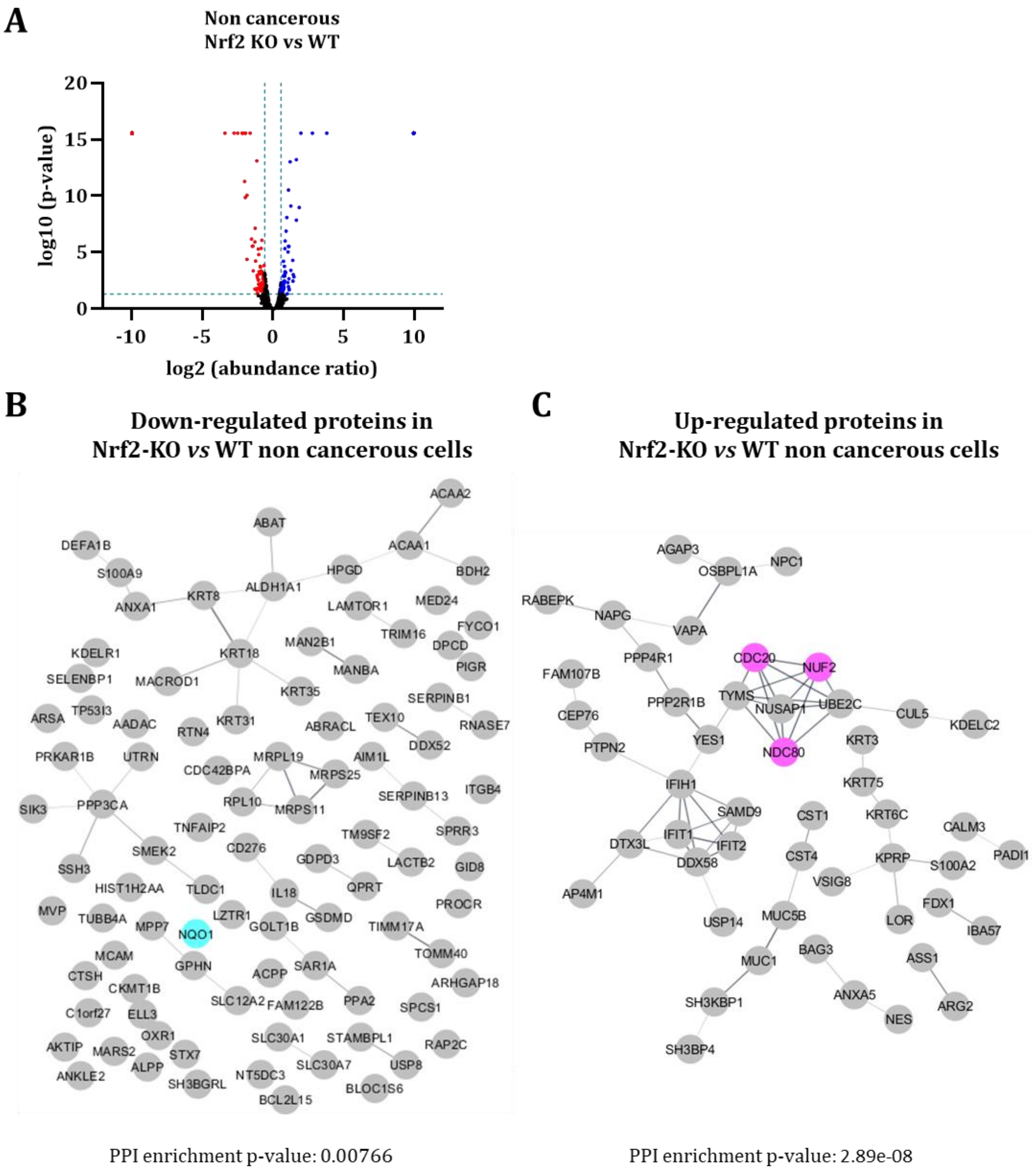

Figure S3. **Global proteomics analysis of the gastric non-cancerous cell line (HFE-145).** (**A**) Volcano plot showing p-values (−log10) *versus* abundance ratio (log2) in Nrf2-KO compared to WT cells. (**B-C**) STRING networks of (B) down-regulated proteins (p-value <0.05; abundance ratio <0.65) and (C) up-regulated proteins (p-value <0.05; abundance ratio >1.5). Blue nodes show proteins related to cellular redox homeostasis and pink nodes show proteins involved in cell cycle regulation.
PPI: protein-protein interaction.

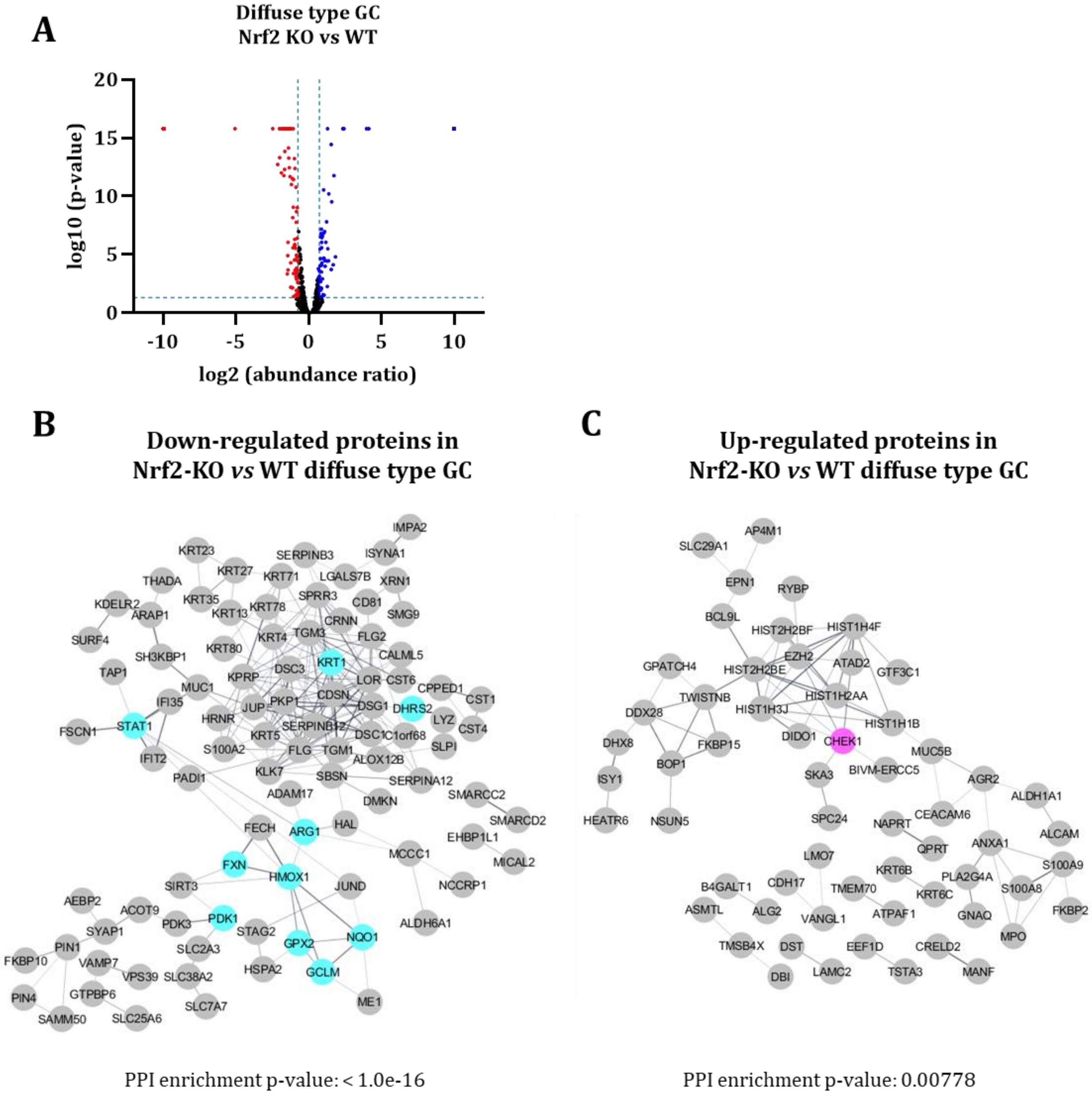

Figure S4. Global proteomics analysis of the diffuse type gastric cancer cell line (AGS). (**A**) Volcano plot showing p-values (−log10) *versus* abundance ratio (log2) in Nrf2-KO compared to WT cells. (**B-C**) STRING networks of (B) down-regulated proteins (p-value <0.05; abundance ratio <0.65) and (C) up-regulated proteins (p-value <0.05; abundance ratio >1.5). Blue nodes show proteins related to cellular redox homeostasis and pink nodes show proteins involved in cell cycle regulation.
PPI: protein-protein interaction.

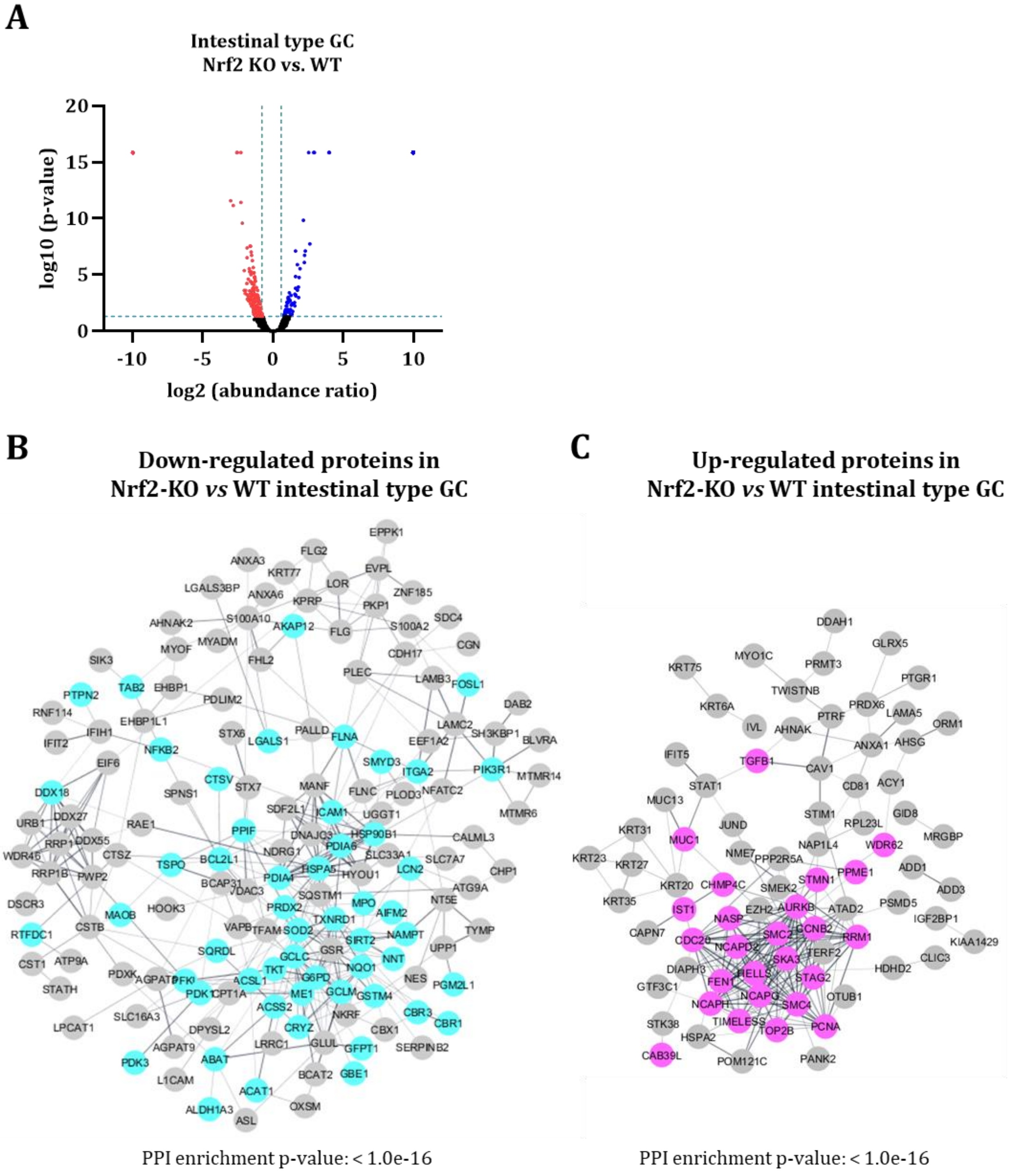

Figure S5. **Global proteomics analysis of the intestinal type gastric cancer cell line (MKN74).** (**A**) Volcano plot showing p-values (−log10) *versus* abundance ratio (log2) in Nrf2-KO compared to WT cells. (**B-C**) STRING networks of (B) down-regulated proteins (p-value <0.05; abundance ratio <0.65) and (C) up-regulated proteins (p-value <0.05; abundance ratio >1.5). Blue nodes show proteins related to cellular redox homeostasis and pink nodes show proteins involved in cell cycle regulation.
PPI: protein-protein interaction.

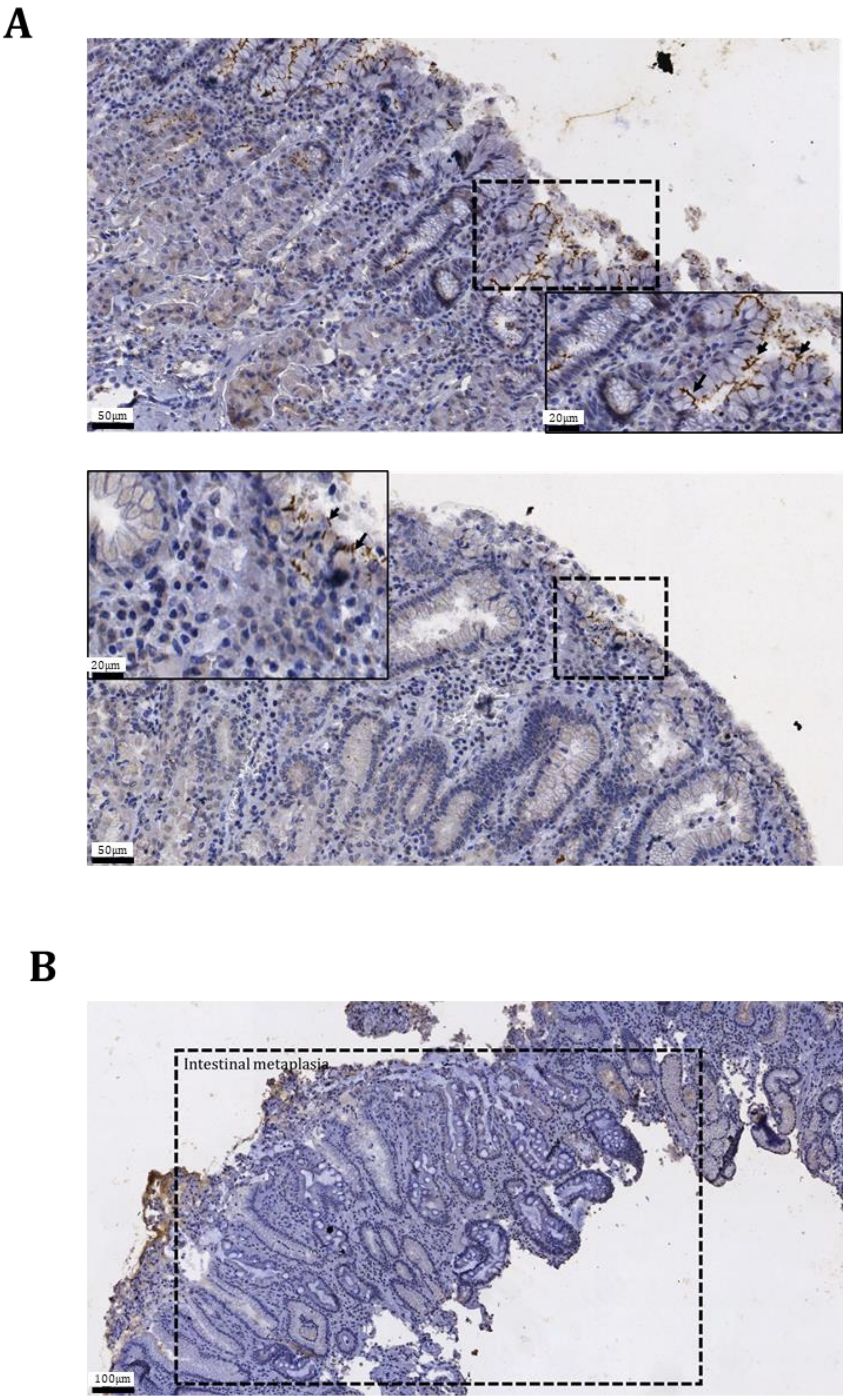

Figure S6. Immunohistochemistry to detect *H. pylori* in infected human biopsies. (**A**) The presence of the bacterium was detected in all infected-samples and depicted by the black arrows in the representative pictures. (**B**) Some *H. pylori*-infected tissues showed intestinal metaplasia area, characteristic of infection with this bacterium.

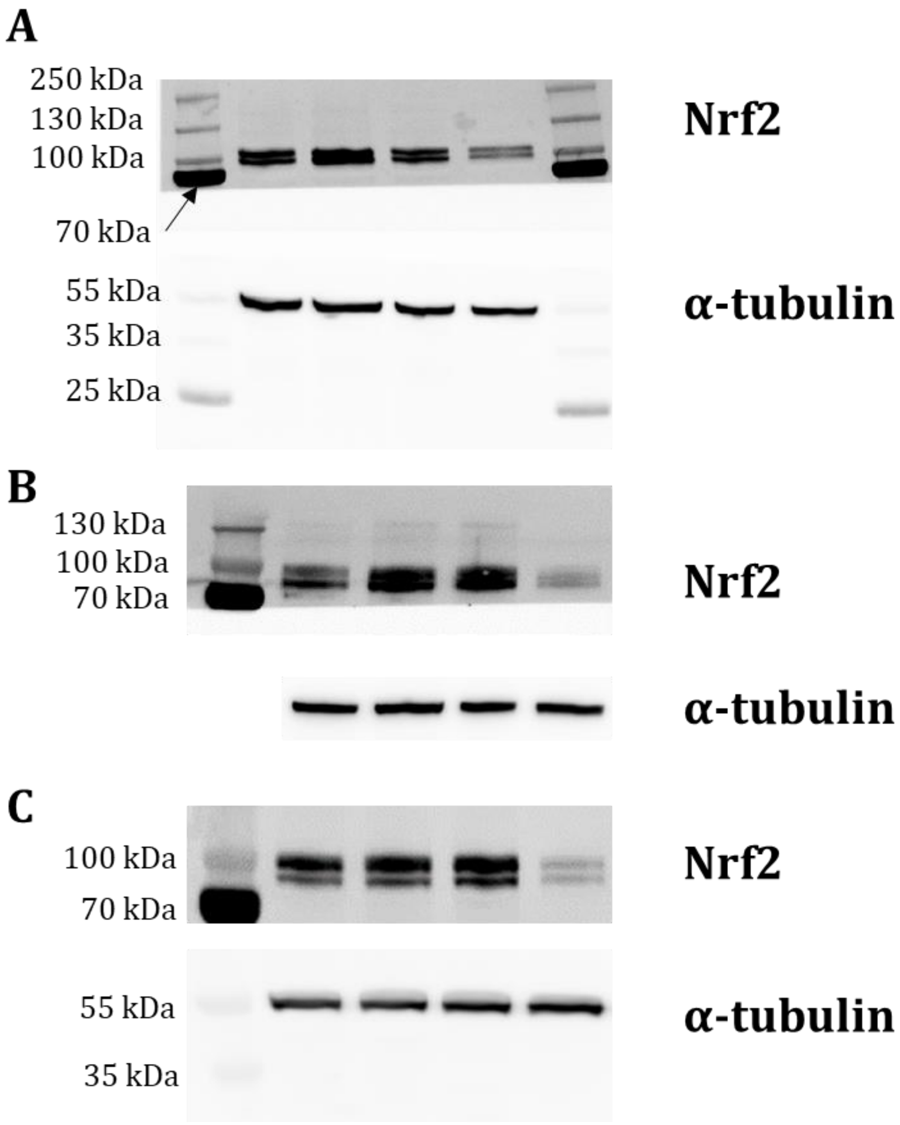

Figure S7. Raw blot of the western-blot presented in Figure 1 (**A**) Non-cancerous HFE-145 cell line (**B**) Diffuse type GC AGS (**C**) Intestinal type GC MKN74.

File S1: SUPPLEMENTARY MATERIAL AND METHODS

Differential proteomic analysis

<u>Sample preparation and protein digestion</u>

Protein samples were solubilized in Laemmli buffer and 2 µg per sample were deposited onto SDS-PAGE gel for concentration and cleaning purpose. Separation was stopped once proteins have entered resolving gel. After colloidal blue staining, bands were cut out from the SDS-PAGE gel and subsequently cut in 1 mm x 1 mm gel pieces. Gel pieces were destained in 25 mM ammonium bicarbonate 50% ACN, rinsed twice in ultrapure water and shrunk in ACN for 10 min. After ACN removal, gel pieces were dried at room temperature, covered with the trypsin solution (10 ng/µl in 50 mM NH_4HCO_3), rehydrated at 4 °C for 10 min, and finally incubated overnight at 37 °C. Spots were then incubated for 15 min in 50 mM NH_4HCO_3 at room temperature with rotary shaking. The supernatant was collected, and an H_2O/ACN/HCOOH (47.5:47.5:5) extraction solution was added onto gel slices for 15 min. The extraction step was repeated twice. Supernatants were pooled and dried in a vacuum centrifuge. Digests were finally solubilized in 0.1% HCOOH.

<u>nLC-MS/MS analysis and Label-Free Quantitative Data Analysis</u>

Peptide mixture was analyzed on a Ultimate 3000 nanoLC system (Dionex, Amsterdam, The Netherlands) coupled to a Electrospray Orbitrap Fusion™ Lumos™ Tribrid™ Mass Spectrometer (Thermo Fisher Scientific, San Jose, CA). Ten microliters of peptide digests were loaded onto a 300-µm-inner diameter x 5-mm C_{18} PepMap™ trap column (LC Packings) at a flow rate of 10 µL/min. The peptides were eluted from the trap column onto an analytical 75-mm id x 50-cm C18 Pep-Map column (LC Packings) with a 4–27.5% linear gradient of solvent B in 105 min (solvent A was 0.1% formic acid and solvent B was 0.1% formic acid in 80% ACN) followed by a 10 min gradient from 27.5% to 40% solvent B. The separation flow rate was set at 300 nL/min. The separation flow rate was set at 300 nL/min. The mass spectrometer operated in positive ion mode at a 2-kV needle voltage. Data were acquired using Xcalibur 4.4 software in a data-dependent mode. MS scans (*m/z* 375-1500) were recorded in the Orbitrap at a resolution of R = 120 000 (@ m/z 200) and an AGC target of 4×10^5 ions collected within 50 ms. Dynamic exclusion was set to 60 s and top speed fragmentation in HCD mode was performed over a 3 s cycle. MS/MS scans were collected in the Ion Trap with a maximum injection time of 35 ms. Only +2 to +7 charged ions were selected for fragmentation. Other settings were as follows: no sheath nor auxiliary gas flow, heated capillary temperature, 275 °C; normalized HCD collision energy of 30%, isolation width of 1.6 m/z, AGC target of 2×10^3 and normalized AGC target of 20%. Monoisotopic precursor selection (MIPS) was set to Peptide and an intensity threshold was set to 5×10^3.

<u>Database search and results processing</u>

Data were searched by SEQUEST through Proteome Discoverer 2.4 (Thermo Fisher Scientific Inc.) against an *Homo sapiens* Reference Proteome Set protein database from Uniprot (20,324 entries; version 2020-05). Spectra from peptides higher than 5000 Da or lower than 350 Da were rejected. Search parameters were as follows: mass accuracy of the monoisotopic peptide precursor and peptide fragments was set to 10 ppm and 0.6 Da respectively. Only b- and y-ions were considered for mass calculation. Oxidation of methionines (+16 Da), methionine loss (-131 Da), methionine loss with acetylation (-89 Da) and protein N-terminal acetylation (+42 Da) were considered as variable modifications while carbamidomethylation of cysteines (+57 Da) was considered as fixed modification. Two missed trypsin cleavages were allowed. Peptide validation was performed using Percolator algorithm [1] and only "high confidence" peptides were retained corresponding to a 1% False Positive Rate at peptide level. Peaks were detected and integrated using the Minora algorithm embedded in Proteome Discoverer. Proteins were quantified based on unique peptide intensities. Normalization was performed based on human protein amount. Protein ratios were calculated as the median of all possible pairwise peptide ratios. A t-test was calculated based on background population of peptides or proteins. Quantitative data were considered for proteins quantified by a minimum of two peptides and a statistical p-value lower than 0.05.

1. Käll, L.; Canterbury, J.D.; Weston, J.; Noble, W.S.; MacCoss, M.J. Semi-Supervised Learning for Peptide Identification from Shotgun Proteomics Datasets. *Nat Methods* **2007**, *4*, 923–925, doi:10.1038/nmeth1113.
